# High HbA1c is associated with decreased 6-month survival and poor outcomes after out-of-hospital cardiac arrest: a retrospective cohort study

**DOI:** 10.1186/s13049-020-00782-1

**Published:** 2020-09-03

**Authors:** Junhaeng Lee, Joo Suk Oh, Jong Ho Zhu, Sungyoup Hong, Sang Hyun Park, Ji Hoon Kim, Hyungsoo Kim, Mingu Seo, Kiwook Kim, Doo Hyo Lee, Hyun Ho Jung, Jungtaek Park, Young Min Oh, Semin Choi, Kyoung Ho Choi

**Affiliations:** 1 Department of Emergency Medicine, Uijeongbu St. Mary’s Hospital, College of Medicine, The Catholic University of Korea, Uijeongbu-si, Republic of Korea; 2 Department of Emergency Medicine, Eunpyeong St. Mary’s Hospital, College of Medicine, The Catholic University of Korea, Seoul, Republic of Korea; 3 Department of Emergency Medicine, Daejeon St. Mary’s Hospital, College of Medicine, The Catholic University of Korea, Daejeon, Republic of Korea; 4 Department of Emergency Medicine, Yeouido St. Mary’s Hospital, College of Medicine, The Catholic University of Korea, Seoul, Republic of Korea; 5 Department of Emergency Medicine, Bucheon St. Mary’s Hospital, College of Medicine, The Catholic University of Korea, Bucheon-si, Republic of Korea

## Abstract

**Background:**

To evaluate the associations between glycated hemoglobin (HbA1c) at admission and 6-month mortality and outcomes after out-of-hospital cardiac arrest (OHCA) treated by hypothermic targeted temperature management (TTM).

**Methods:**

This single-center retrospective cohort study included adult OHCA survivors who underwent hypothermic TTM from December 2011 to December 2019. High HbA1c at admission was defined as a level higher than 6%. Poor neurological outcomes were defined as cerebral performance category scores of 3–5. The primary outcome was 6-month mortality. The secondary outcome was the 6-month neurological outcome. Descriptive statistics, log-rank tests, and multivariable regression modeling were used for data analysis.

**Results:**

Of the 302 patients included in the final analysis, 102 patients (33.8%) had HbA1c levels higher than 6%. The high HbA1c group had significantly worse 6-month survival (12.7% vs. 37.5%, *p* < 0.001) and 6-month outcomes (89.2% vs. 73.0%, *p* = 0.001) than the non-high HbA1c group. Kaplan-Meier analysis and the log-rank test showed that the survival time was significantly shorter in the patients with HbA1c > 6% than in those with HbA1c ≤6%. In the multivariable logistic regression analysis, HbA1c > 6% was independently associated with 6-month mortality (OR 5.85, 95% CI 2.26–15.12, p < 0.001) and poor outcomes (OR 4.18, 95% CI 1.41–12.40, p < 0.001).

**Conclusions:**

This study showed that HbA1c higher than 6% at admission was associated with increased 6-month mortality and poor outcomes in OHCA survivors treated with hypothermic TTM. Poor long-term glycemic management may have prognostic significance after cardiac arrest.

## Background

Approximately 25,000 Koreans experience out-of-hospital cardiac arrest (OHCA) each year [1]. OHCA is a leading cause of death in high-to-middle income countries [2]. Despite improvements in resuscitation and postcardiac arrest care, the rates of mortality and permanent brain damage in OHCA patients remain high [3, 4].

One meta-analysis demonstrated that diabetes mellitus (DM) is associated with lower survival to discharge and poor neurologic outcome in OHCA patients [5]. DM, a heterogeneous condition characterized by abnormal insulin control, can lead to both microvascular and macrovascular diseases via multiple pathways and is associated with a set of well-known risk factors for OHCA, such as atherosclerotic coronary heart disease [6]. Additionally, elevated blood glucose levels after the return of spontaneous circulation (ROSC) correlate with worse outcomes following OHCA [7, 8]. However, the relationships between the glucose level and outcomes after cardiac arrest remain debatable [9].

The level of glycated hemoglobin (HbA1c) reflects the long-term glycemic status over the preceding 4–12 weeks [10–12]. HbA1c is increasingly used in clinical practice because of the test’s convenience and reliability. It has been found to be more predictive of cardiovascular disease complications than single, time-sensitive glucose levels in patients with and without DM [13]. Patients with elevated HbA1c tend to suffer from early neurological deterioration or new ischemic lesions after acute ischemic stroke [14, 15]. Meanwhile, hypothermia alters glucose homeostasis, interfering with glucose control and insulin therapy [16]. Moreover, increased glycemic variability after cardiac arrest is associated with a poor outcome [7, 17]. It can be assumed that maintaining glycemic control in patients undergoing hypothermic targeted temperature management (TTM) will be challenging in those with poor baseline glycemic control. However, few studies have investigated the association of long-term glycemic status, as reflected by the HbA1c level, with outcomes in OHCA patients. The purpose of the current study was to investigate the association between HbA1c at the time of admission and 6-month survival and neurological outcomes after OHCA treated with hypothermic TTM.

## Methods

### Study design and setting

This study was approved by the Institutional Review Board of the Catholic University of Korea (UC20RISI0091). The need to obtain informed consent was waived because of the retrospective nature of the study. This retrospective observational cohort study was performed in a single center, Uijeongbu St. Mary’s Hospital, Catholic University of Korea, which is a regional emergency medical center and regional trauma center. The annual emergency department volume is approximately 80,000 visits, providing postcardiac arrest care with hypothermic TTM to more than 45 patients annually.

### Population

Postcardiac arrest (PCAS) patients who had undergone hypothermic TTM were included. Adults (≥18 years old) who maintained ROSC for longer than 20 min were eligible for hypothermic TTM. Patients who did not receive hypothermic TTM and those for whom blood HbA1c levels at admission were not available were excluded from the analysis. Patients did not receive hypothermic TTM for the following reasons: recovery of consciousness after ROSC; past history of irreparable brain damage (cerebral performance categories (CPC) scale 3–4); suspected hemorrhagic shock; suspected intracranial hemorrhage; known terminal illness; and refusal of the patient’s family.

### Measures

The primary outcome was 6-month mortality, while the secondary outcome was the 6-month neurological outcome. Good neurological outcomes were defined as CPC scores of 1–2, while poor neurological outcomes were defined as CPC scores of 3–5. Patient demographics and cardiac arrest variables were collected according to the Utstein style guidelines [18]. We recorded the following baseline clinical data: age, sex, previous medical history (e.g., acute myocardial infarction, previous cardiac arrest, angina, congestive heart failure, hypertension (HTN), DM, lung disease, cerebrovascular accident (CVA), renal disease, liver cirrhosis, malignancy), initial cardiac arrest rhythm (shockable or nonshockable), presence of a witness, bystander cardiopulmonary resuscitation (CPR), and time from collapse to ROSC (anoxic time). We also recorded the following glucose-related variables: HbA1c at admission, initial glucose level, and glucose level variability within 48 h after ROSC. Blood samples for the determination of HbA1c and initial glucose levels were collected immediately after ROSC. Blood sugar tests were performed every hour until 48 h after ROSC. Glucose level variability was calculated as the largest difference between minimum and maximum values during the first 48 h after ROSC (Δ glucose). We collected medical data from the medical database. We obtained the survival time from the National Health Insurance Service database. We obtained the neurologic status of the patients by directly calling the patient’s caregiver 6 months after ROSC. We determined an optimal HbA1c cutoff of 6% for predicting 6-month mortality and the neurologic outcome. The guidelines recommend an HbA1c ≥6.5% to diagnose diabetes and an HbA1c between 5.7 and 6.4% for identifying prediabetes [19–21]. These cutoff values for HbA1c were derived in part from outpatient studies and were based on populations of those not acutely ill at the time of testing. Silverman et al. suggested that in an acute-care setting such as the emergency department, an HbA1c of 5.7% was the optimal screening cutoff for prediabetes, and 6% was the optimal screening cutoff for diabetes [22]. The South Korean health system does not permit the withdrawal of life-sustaining treatment (WLST) from patients; therefore, we did not use the collected data in the present study to make any decisions regarding the withdrawal of life support from any patient. However, do-not-resuscitate (DNR) orders are legal and socially acceptable. Therefore, if the family did not want to escalate treatment after the prognosis was determined, it was not performed.

### TTM protocol

The postcardiac arrest care performed on the study subjects was based on the CPR guidelines from the American Heart Association and Korean CPR Association [23, 24]. To reach the target temperature, induction was started immediately after ROSC with ice packs and an automatic surface cooling device using hydrogel pads (Arctic Sun 5000, Medivance Inc., Louisville, CO, USA); the target temperature was 33 °C for 24 h, followed by 12 h of rewarming at the rate of 0.25 °C per hour. After reaching the target rewarming temperature of 36.8 °C, we continued normothermic TTM for an additional 36 h. We continuously infused midazolam (0.04–0.2 mg/kg/hr) and rocuronium (0.3–0.6 mg/kg/hr) to control shivering during the hypothermic period. All patients were admitted to the intensive care unit and received standard intensive care. The following clinical parameters were used to achieve hemodynamic optimization, ventilator management, and glucose management: SaO2 of 94 to 96%, PaCO2 of 35 to 45 mmHg, mean arterial pressure greater than or equal to 70 mmHg, urine output greater than or equal to 0.5 mL/kg per hour, and glucose level of 144 to 180 mg/dL. In addition, we monitored amplitude-integrated electroencephalography. If there was evidence of epileptic discharges, we administered anti-epileptic drugs (valproic acid, levetiracetam, clonazepam).

### Data analysis

This study was conducted in accordance with the Strengthening of the Reporting of Observational Studies in Epidemiology statement [25].

As there were no preliminary data, we assumed that the group with HbA1c ≤6% would have twice as many patients as the group with HbA1c > 6%, according to our clinical experience. We calculated the sample size to detect a difference between survival rates of 0.3 (HbA1c ≤6%) and 0.15 (HbA1c > 6%). Finally, a total of 302 subjects were needed to achieve a power of 80% at a significance level of 0.01.

We present categorical variables as absolute numbers with percentages; these variables were compared with the chi-square test or Fisher’s exact test. Continuous variables are presented as median values with interquartile ranges (IQR) and were compared with the Mann-Whitney U test because the continuous variables had nonparametric distributions.

We used the log-rank test to compare the survival rates between the group with HbA1c ≤6% and the group with HbA1c > 6%. Kaplan-Meier analysis survival curves were generated to analyze 6-month survival.

A multivariate logistic regression analysis was used to assess the effects of the predictors. All variables with a significance level less than 0.01 according to univariate analysis and cardiac arrest characteristics (nonshockable rhythm, absence of witness, no bystander CPR, time to ROSC) were included in the multivariate logistic regression model. The goodness of fit of the model was evaluated by the Hosmer-Lemeshow test. The results of the logistic regression analysis are presented as odds ratios (ORs) and 95% confidence intervals (CIs). All of the statistical analyses were performed using MedCalc version 19.1.5. (MedCalc software, Mariakerke, Belgium). *P* values < 0.01 were considered statistically significant (two-sided).

## Results

**Fig. 1:**
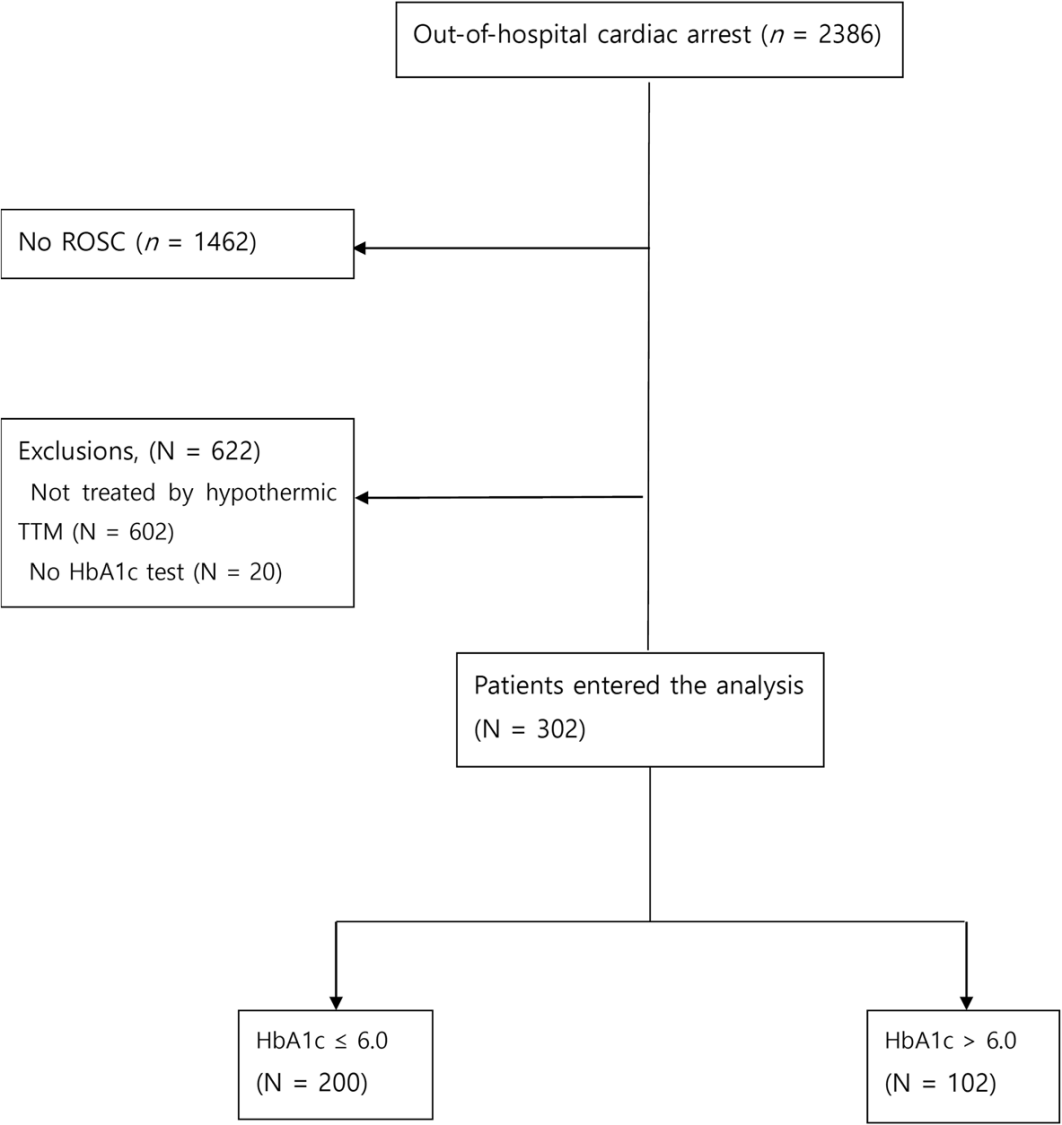
Study flow diagram. ROSC = return of spontaneous circulation, HbA1c = glycated hemoglobin

Out of a total of 2386 OHCA patients who were admitted from December 2011 to December 2019, 1462 did not achieve ROSC. Of the remaining 924 patients, 622 patients were excluded because they did not receive hypothermic TTM or did not have available HbA1c results. Finally, 302 patients were included in the analysis. Of these patients, 102 patients (33.8%) had HbA1c levels higher than 6% (Fig. 1). There were no missing data on survival or neurological outcomes.

Patients with HbA1c > 6% were older than those with HbA1c ≤6% (*p* < 0.001). They also had higher incidences of HTN (*p* < 0.001), CVA (*p* = 0.004) and DM (*p* < 0.001). Glucose levels were also significantly higher in the patients with HbA1c > 6% than in the patients with HbA1c ≤6% (*p* < 0.001). Glucose level variability was measured in the 237 patients who survived for 48 h, and patients with HbA1c > 6% had a significantly higher glucose level variability than the patients with HbA1c ≤6% (*p* = 0.004) (Table 1).

**Table 1:** Demographic and clinical characteristics

	Total (*n* = 302)	HbA1c ≤6% (*n* = 200)	HbA1c > 6% (*n* = 102)	*p*
Demographic characteristics				
Male, *n* (%)	207 (68.5)	139 (69.5)	68 (66.7)	0.62
Age, years, median (IQR)	61 (49–72)	57.0 (45–70)	67.5 (56–75)	< 0.001
Underlying disease, *n* (%)				
Acute myocardial infarction	17 (5.6)	9 (4.5)	8 (7.8)	0.23
Previous cardiac arrest	1 (0.3)	1 (0.5)	0 (0.0)	1.00
Angina	34 (11.3)	20 (10.0)	14 (13.7)	0.34
Congestive heart failure	20 (6.6)	9 (4.5)	11 (10.8)	0.05
Hypertension	123 (40.7)	64 (32.0)	59 (57.8)	< 0.001
Cerebrovascular accident	23 (7.6)	9 (4.5)	14 (13.7)	0.004
Diabetes mellitus	75 (24.8)	17 (8.5)	58 (56.9)	< 0.001
Lung disease	29 (9.6)	20 (10.0)	9 (8.8)	0.84
Neurological disease	21 (7.0)	12 (6.0)	9 (8.8)	0.35
Renal disease	27 (8.9)	12 (6.0)	15 (14.7)	0.02
Liver cirrhosis	5 (1.7)	4 (2.0)	1 (1.0)	0.67
Malignancy	17 (5.6)	12 (6.0)	5 (4.9)	0.80
Cardiac arrest characteristics				
Shockable rhythm, *n* (%)	199 (65.9)	127 (63.5)	72 (70.6)	0.25
Witnessed arrest, *n* (%)	114 (37.7)	81 (40.5)	33 (32.4)	0.21
Bystander CPR, *n* (%)	147 (48.7)	101 (50.5)	46 (45.1)	0.40
Anoxic time, min, median (IQR)	30 (17–42)	30 (17–43)	30 (17–40)	0.88
Glucose-related variables				
Initial glucose level, mg/dL, median (IQR)	240 (70–521)	227 (72–414)	274 (70–648)	< 0.001
Glucose level variability within 48 h, Δ glucose, median (IQR)	140 (90–208) (*n* = 237)	135 (81.5–187) (*n* = 164)	154 (120.8–249.8) (*n* = 73)	0.004

Continuous variables are expressed as medians (interquartile ranges). *HbA1c* glycated hemoglobin, *IQR* interquartile range, *CPR* cardiopulmonary resuscitation, *ROSC* return of spontaneous circulation, *S100B* calcium-binding protein B

**Fig. 2:**
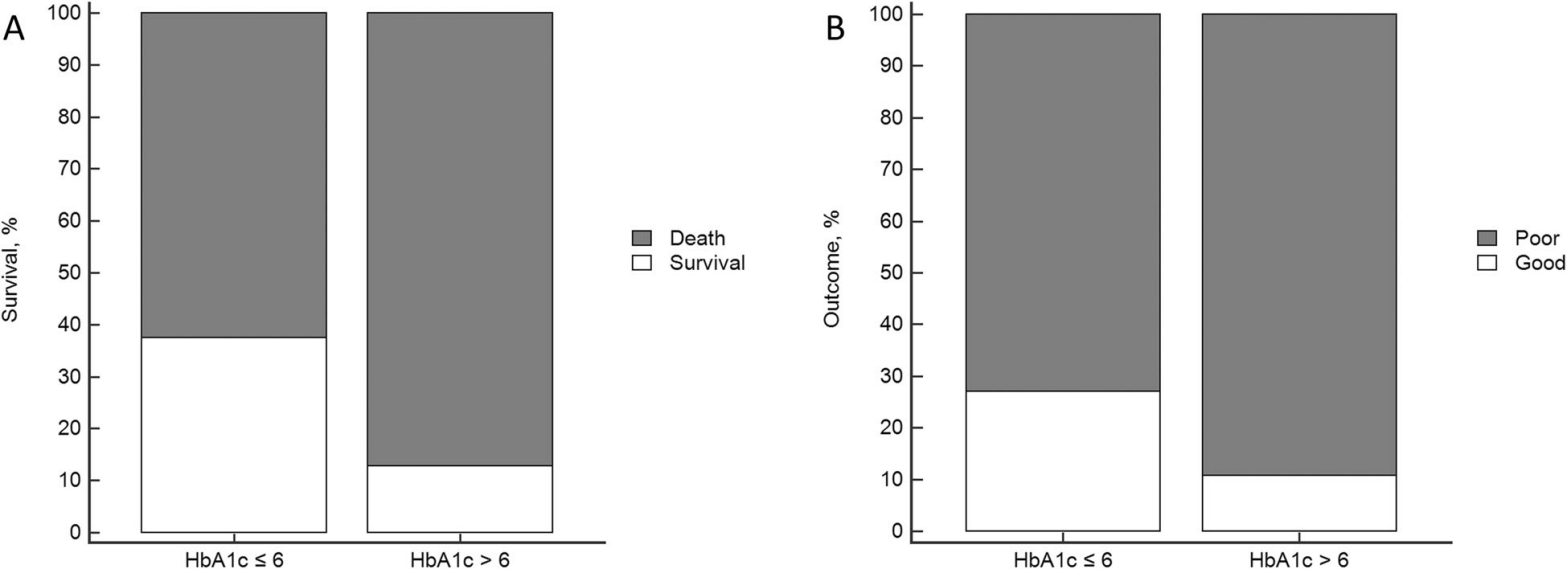
Association of HbA1c with 6-month survival (**a**) and 6-month neurological outcomes (**b**). HbA1c = glycated hemoglobin

Figure 2 shows that HbA1c > 6% was associated with a decreased 6-month survival rate (> 6 12.7% vs. ≤6 37.5%, *p* < 0.001; Fig. 2a). Additionally, HbA1c > 6% was associated with a worse 6-month neurological outcome (> 6 89.2% vs. ≤6 73.0%, *p* = 0.001; Fig. 2b).

**Fig. 3:**
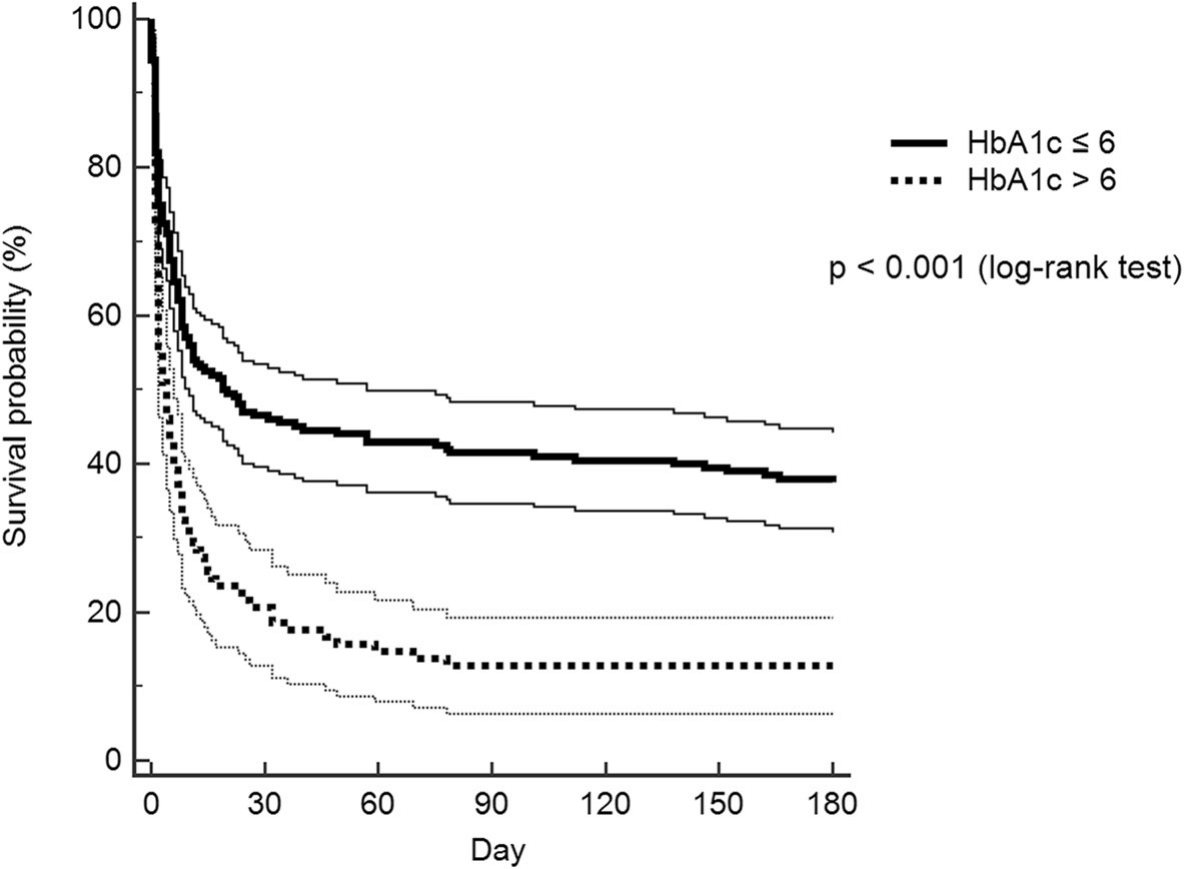
Kaplan-Meier curve demonstrating reduced survival in patients with HbA1c > 6% compared to those with HbA1c ≤6%. HbA1c = glycated hemoglobin

Kaplan-Meier analysis and the log-rank test showed that the survival time was significantly shorter in the group of patients with HbA1c > 6% (30.54 days; 95% CI 19.14–71.68) than in the group of patients with HbA1c ≤6% (79.11 days; 95% CI 67.50–90.71) (Fig. 3). From the multivariable logistic regression, HbA1c > 6% (*p* < 0.001), a nonshockable rhythm (*p* < 0.001), older age (*p* < 0.001), and a longer anoxic time (*p* < 0.001) were significantly associated with 6-month mortality. With regard to the 6-month neurologic outcome, HbA1c > 6% (*p* = 0.009), a nonshockable rhythm (*p* < 0.001), older age (*p* < 0.001), and a longer anoxic time (*p* < 0.001) were significantly associated with a poor neurologic outcome. The glucose level, previous illnesses (HTN, DM, CVA), the absence of a witness, and no bystander CPR were not statistically associated with 6-month mortality or neurological outcomes (Table 2).

**Table 2:** Multivariable logistic regression analysis for 6-month mortality and poor 6-month neurological outcomes

	6-month mortality		Poor 6-month outcome	
	OR (95% CI)	*p*	OR (95% CI)	*p*
HbA1c > 6%	5.846 (2.26–15.12)	< 0.001	4.180 (1.41–12.39)	0.009
Nonshockable rhythm	5.204 (2.62–10.34)	< 0.001	8.878 (4.11–19.16)	< 0.001
Age	1.060 (1.03–1.09)	< 0.001	1.050 (1.02–1.08)	< 0.001
Anoxic time	1.077 (1.05–1.10)	< 0.001	1.072 (1.04–1.10)	< 0.001
Glucose	1.001 (0.10–1.00)	0.70	1.003 (0.10–1.01)	0.12
Hypertension	0.434 (0.21–0.92)	0.03	0.558 (0.25–1.25)	0.16
Diabetes mellitus	0.817 (0.30–2.27)	0.70	0.462 (0.14–1.49)	0.20
Cerebrovascular accident	0.957 (0.29–3.19)	0.94	1.218 (0.29–5.11)	0.79
No witness	2.352 (1.09–5.07)	0.03	2.816 (1.14–6.96)	0.03
No bystander CPR	1.086 (0.53–2.21)	0.82	0.885 (0.40–1.98)	0.77

*OR* odds ratio, *CI* confidence interval, *HbA1c* glycated hemoglobin, *ROSC* return of spontaneous circulation, *CPR* cardiopulmonary resuscitation

## Discussion

This retrospective cohort study demonstrated that an HbA1c level higher than 6% in patients with OHCA was associated with increased 6-month mortality and a poor neurologic outcome after treatment with hypothermic TTM.

HbA1c measurement has become an integral tool for the diagnosis and management of DM. It also serves as a surrogate marker of glycemic control and is a key risk indicator for diabetes-associated microvascular and macrovascular complications and mortality. One study showed that patients with HbA1c ≥6.5% had a significant 2-fold higher risk of sudden cardiac arrest than those with lower levels, even after controlling for multiple cardiovascular disease risk factors [26]. Kim et al. found that an HbA1c level greater than 8% at admission was significantly independently associated with early neurologic deterioration in patients with acute atrial fibrillation-related ischemic stroke [13]. Another study suggested that elevated HbA1c levels higher than 6.5% were associated with new ischemic lesions in patients with acute ischemic stroke [14].

There are several possible explanations for the association between HbA1c and outcomes. One study reported that the HbA1c level at ICU admission was associated with the progression of organ dysfunction and mortality in patients with sepsis [27]. The HbA1c level reflects a patient’s premorbid glycemic state during the preceding 3 months [10]. A chronic hyperglycemic state can damage the endothelial glycocalyx; this is considered to be the primary mechanism responsible for vascular complications in patients with DM. Degradation of the glycocalyx alters endothelial barrier permeability and may thus cause damage to the microcirculation, which contributes to organ dysfunction [28–30]. Another study showed that HbA1c was a significant predictor of major adverse cardiac events after acute myocardial infarction (AMI) in nondiabetic patients [31]. Glycemic control may contribute to the damage to vascular structures caused by a poor glycemic status. The mechanisms might include severe coronary endothelial dysfunction resulting from increased oxidative stress caused by high glucose levels, increased platelet adhesion that promotes venous thrombosis and enhanced inflammatory responses that cause the progression of atherosclerosis or vascular injury [32–35]. Furthermore, poor glycemic control can lead to an increased formation of advanced glycation end products, which can cause severe vascular damage [32]. Therefore, HbA1c, which reflects metabolic control and embedded ongoing vascular injury or atherosclerosis, can be considered a reliable indicator of adverse outcomes in nondiabetic populations after AMI [31]. It should be noted that glucose level variability was greater in patients with high HbA1c than in those with normal HbA1c. Previous studies have shown that hypothermia impairs glucose homeostasis, leading to increased glucose level variability [7, 16, 17]. Moreover, increased glucose level variability is known to be a predictor of postcardiac arrest mortality and neurological outcomes [7, 17]. A high HbA1c level can lead to difficulty maintaining glycemic control under hypothermic TTM. Thus, clinicians should be aware of the HbA1c level and make an effort to reduce glucose level variability in patients with HbA1c levels higher than 6%.

This study should be interpreted with consideration of the following limitations. First, the HbA1c level can be influenced by other factors. The HbA1c level is affected by the blood glucose concentration, the duration of red blood cell (RBC) exposure to varying concentrations, and RBC quantity. In adults, HbA1c is often falsely low in patients with conditions that reduce the number of glycosylated RBCs, such as hemolysis, splenomegaly, chronic kidney disease, cirrhosis, and hemorrhage. Alternately, HbA1c levels are elevated in patients with conditions that result in decreased RBC turnover, such as iron- or vitamin B12-deficiency anemia [36–38]. Second, this study included patients with DNR orders. Although WLST is not allowed in South Korea, this study did not exclude patients with DNR orders, and such orders could have influenced decisions regarding withholding advanced treatment. Third, this study reflects the experience at a single institution, which may limit the generalizability of the results. However, our study included a large sample size over the course of 8 years. Fourth, this study was a retrospective study, which may have caused inevitable selection bias.

## Conclusions

This study showed that an HbA1c level higher than 6% at the time of admission was associated with higher 6-month mortality and poor neurological outcomes in OHCA patients treated with hypothermic TTM. Long-term poor glycemic status before cardiac arrest might be related to the deterioration of the condition of the patients and poor glycemic control during hypothermic TTM. The HbA1c level can be considered a better indicator than the glucose level to facilitate the early detection of patients with potential adverse prognoses after OHCA. Additional prospective, multicenter studies are needed to confirm these findings.

## Data Availability

The datasets used and/or analyzed during the current study are available from the corresponding author on reasonable request.

## Abbreviations

OHCA: out-of-hospital cardiac arrest
DM: diabetes mellitus
ROSC: return of spontaneous circulation
HbA1c: glycated hemoglobin
TTM: targeted temperature management
PCAS: post cardiac arrest syndrome
CPC: cerebral performance category
HTN: hypertension
CVA: cerebrovascular accident
CPR: cardiopulmonary resuscitation
WLST: withdrawal of life-sustaining treatment
DNR: do-not-resuscitate
IQR: interquartile ranges
OR: odds ratio
CI: confidence interval
AMI: acute myocardial infarction
RBC: red blood cell

## References

[1] Korea Centers for Disease Control and Prevention. EMS-based database for medical record review of out-of-hospital cardiac arrest and mass casualty incident injury. Cheongju: Korea Centers for Disease Control and Prevention; 2011.

[2] Berdowski J, Berg RA, Tijssen JG, Koster RW (2010). Global incidences of out-of-hospital cardiac arrest and survival rates: systematic review of 67 prospective studies. Resuscitation..

[3] Dragancea I, Horn J, Kuiper M (2015). Neurological prognostication after cardiac arrest and targeted temperature management 33°C versus 36°C: results from a randomised controlled clinical trial. Resuscitation..

[4] Kirkegaard H, Rasmussen BS, de Haas I (2016). Time-differentiated target temperature management after out-of-hospital cardiac arrest: a multicentre, randomised, parallel-group, assessor-blinded clinical trial (the TTH48 trial): study protocol for a randomised controlled trial. Trials..

[5] Voruganti DC, Chennamadhavuni A, Garje R (2018). Association between diabetes mellitus and poor patient outcomes after out-of-hospital cardiac arrest: a systematic review and meta-analysis. Sci Rep.

[6] Kucharska-Newton AM, Couper DJ, Pankow JS (2010). Diabetes and the risk of sudden cardiac death, the atherosclerosis risk in communities study. Acta Diabetol.

[7] Daviaud F, Dumas F, Demars N (2014). Blood glucose level and outcome after cardiac arrest: insights from a large registry in the hypothermia era. Intens Care Med.

[8] Russo JJ, James TE, Hibbert B (2016). Hyperglycaemia in comatose survivors of out-of-hospital cardiac arrest. Eur Heart J Acute Cardiovasc Care.

[9] Losert H, Sterz F, Roine RO (2007). Strict normoglycaemic blood glucose levels in the therapeutic management of patients within 12 h after cardiac arrest might not be necessary. Resuscitation..

[10] Nathan DM, Turgeon H, Regan S (2007). Relationship between glycated haemoglobin levels and mean glucose levels over time. Diabetologia..

[11] Hempe JM, Gomez R, McCarter RJ, Chalew SA (2002). High and low hemoglobin glycation phenotypes in type 1 diabetes: a challenge for interpretation of glycemic control. J Diabetes Complicat.

[12] Nathan DM, Singer DE, Hurxthal K, Goodson JD (1984). The clinical information value of the glycosylated hemoglobin assay. N Engl J Med.

[13] Fox CS, Golden SH, Anderson C (2015). Update on prevention of cardiovascular disease in adults with type 2 diabetes mellitus in light of recent evidence :a scientific statement from the American Heart Association and the American Diabetes Association. Circulation..

[14] Kim JS, Kim RY, Cha JK (2017). Pre-stroke glycemic control is associated with early neurologic deterioration in acute atrial fibrillationrelated ischemic stroke. eNeurologicalSci..

[15] Braemswig TB, Nolte CH, Fiebach JB, Usnich T (2017). Early new ischemic lesions located outside the initially affected vascular territory appear more often in stroke patients with elevated glycated hemoglobin (HbA1c). Front Neurol.

[16] Polderman KH (2009). Mechanisms of action, physiological effects, and complications of hypothermia. Crit Care Med.

[17] Cueni-Villoz N, Devigili A, Delodder F (2011). Increased blood glucose variability during therapeutic hypothermia and outcome after cardiac arrest. Crit Care Med.

[18] Langhelle A, Nolan J, Herliz J (2005). Recommended guidelines for reviewing, reporting, and conducting research on post-resuscitation care: the Utstein style. Resuscitation..

[19] Edelman D, Olsen MK, Dudley TK (2004). Utility of hemoglobin A1C in predicting diabetes risk. J Gen Intern Med.

[20] Pradhan AD, Rifai N, Buring JE (2007). Hemoglobin A1c predicts diabetes but not cardiovascular disease in nondiabetic women. Am J Med.

[21] American Diabetes Association (2006). Diagnosis and classification of diabetes mellitus. Diabetes Care.

[22] Silverman RA, Smith K, Thakker U (2011). Hemoglobin A1c as a screen for previously undiagnosed prediabetes and diabetes in an acute-care setting. Diabetes Care.

[23] Clifton WC, Michael WD, Ericka LF (2015). Part 8: post-cardiac arrest care: 2015 Amerian heart association guidelines update for cardiopulmonary resuscitation and emergency cardiovascular care. Circulation..

[24] Kim YM, Park KN, Choi SP (2016). Part 4: post-cardiac arrest care: 2015 Korean guidelines for cardiopulmonary resuscitation. Clin Exp Emerg Med.

[25] von Elm E, Altman DG, Egger M, Pocock SJ, Gøtzsche PC, Vandenbroucke JP, for the STROBE initiative (2008). The strengthening the reporting of observational studies in epidemiology (STROBE) statement: guidelines for reporting observational studies. J Clin Epidemiol.

[26] Patel RB, Moorthy MV, Chiuve SE (2017). Hemoglobin A1c levels and risk of sudden cardiac death: a nested case-cohort study. Heart Rhythm.

[27] Lee YS, Min KH, Lee SY (2019). The value of glycated hemoglobin as predictor of organ dysfunction in patients with sepsis. PLoS One.

[28] Singh A, Friden V, Dasgupta I (2011). High glucose causes dysfunction of the human glomerular endothelial glycocalyx. Am J Physiol Renal Physiol.

[29] Perrin RM, Harper SJ, Bates DO (2007). A role for the endothelial glycocalyx in regulating microvascular permeability in diabetes mellitus. Cell Biochem Biophy.

[30] Salmon AH, Satchell SC (2012). Endothelial glycocalyx dysfunction in disease: albuminuria and increased microvascular permeability. J Pathol.

[31] Chen CL, Yen HT, Lin CS (2017). Glycated hemoglobin level is an independent predictor of major adverse cardiac events after nonfatal acute myocardial infarction in nondiabetic patients. A retrospective observational study. Medicine.

[32] Woodman RJ, Chew GT, Watts GF (2005). Mechanisms, significance and treatment of vascular dysfunction in type 2 diabetes mellitus. Drugs..

[33] Ferreiro GF, Angiolillo DJ (2011). Diabetes and antiplatelet therapy in acute coronary syndrome. Circulation..

[34] Pickup JC, Mattock MB, Chusney GD (1997). NIDDM as a disease of the innate immune system: association of acute-phase reactants and interleukin-6 with metabolic syndrome X. Diabetologia..

[35] Fukuhara M, Matsumura K, Wakisaka M (2007). Hyperglycemia promotes microinflammation as evaluated by C-reactive protein in the very elderly. Intern Med.

[36] Koga M (2014). Glycated albumin: clinical usefulness. Clin Chim Acta.

[37] International Expert Committee (2009). International expert committee report on the role of the A1c assay in the diagnosis of diabetes. Diabetes Care.

[38] World Health Organization. Use of glycated haemoglobin (HbA1c) in the diagnosis of diabetes mellitus. Abbreviated report of a WHO consultation. Geneva, World Health Organization, 2011. http://www.who.int/diabetes/publications/report-hba1c_2011.pdf Accessed 11 May 2020. 26158184

